# A fishy situation – expanding the differential diagnosis of the fish-mouth papilla

**DOI:** 10.1055/a-2751-9555

**Published:** 2026-01-08

**Authors:** Omar El Ouarzadi, Reda Goudrar, Marc-André Smith, Marcel Tomaszewski

**Affiliations:** 1 12368 Faculté de Médecine, Université de Montréal, Montreal, Canada; 2 67120 Department of Microbiology and Infectiology, Hôpital Sacré-Cœur de Montréal, Montreal, Canada; 3 67120 Department of Gastroenterology, Hôpital Sacré-Cœur de Montréal, Montreal, Canada

Hydatid disease (echinococcosis) is a parasitic infection that presents with hepatic cysts and pulmonary involvement. Hepatic hydatid disease can lead to an obstruction of the biliary tree in the case of cyst rupture 1 . This case report provides endoscopic images of a hepatic hydatid cyst mimicking the fish-mouth appearance of papilla, usually pathognomonic for main duct intraductal papillary mucinous neoplasm (MD-IPMN 2 ).

**Fig. 1:**
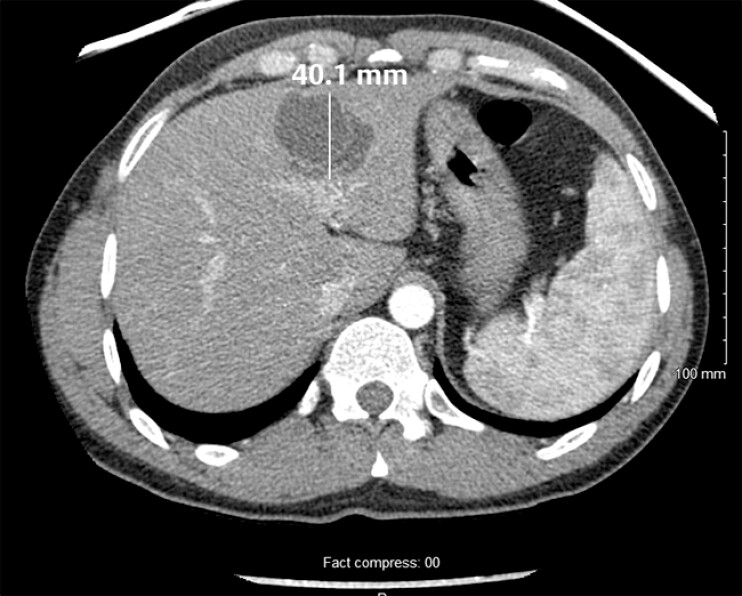
A CT scan showed a multi-lobulated, septated, calcified lesion in the inferior left liver, suggestive of a hepatic cyst. CT, computed tomography.

A 35-year-old man, from North Africa, presented with acute on chronic epigastric pain. Initial work-up revealed increased alanine aminotransferase (648 IU/L) and total bilirubin (99 μmol/L) levels. A computed tomographic scan showed a multilobulated, septated, calcified lesion in the inferior left liver, suggestive of a hepatic cyst ( Fig. 1 ). He was later lost to follow-up.

**Fig. 2:**
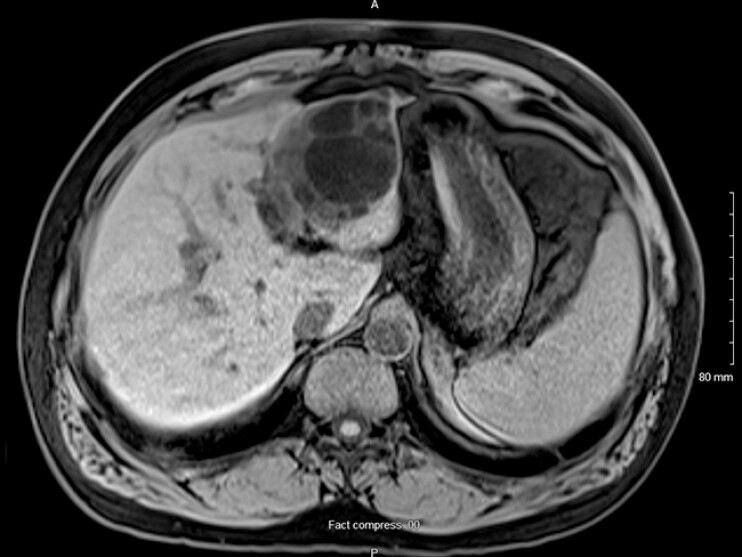
MRI revealed interval growth of the hepatic cyst and left intra-hepatic biliary ductal dilatation. MRI, magnetic resonance imaging.

Nine years later, he returned to the emergency room with work-up confirming cholangitis. MRCP showed poorly defined obstructive filling defects within a slightly dilated bile of 8 mm in diameter ( Fig. 2 ). Interval growth of the hepatic cyst and left intrahepatic biliary ductal dilatation was noted.

**Fig. 3:**
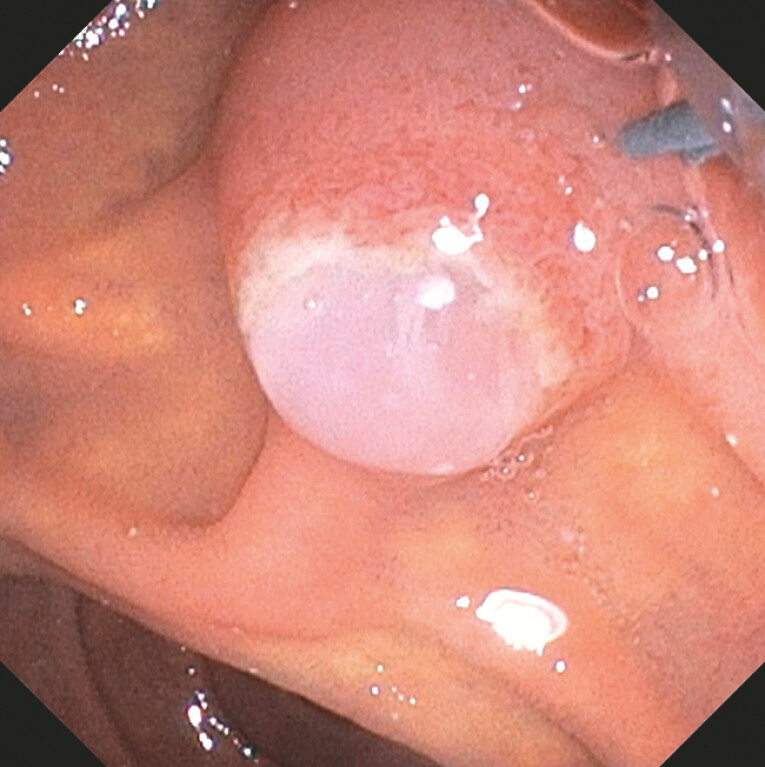
ERCP: a white, soft, mucinous substance protruding from the ampulla, suggestive of a fish-mouth papilla.

ERCP demonstrated a white, soft, mucinous substance protruding from the ampulla ( Video 1 ). The endoscopic images were suggestive of a fish-mouth papilla ( Fig. 3 ). After biliary sphincterotomy, the spontaneous discharge of thick, white membranes occurred.

Fish-mouth papilla appearance of the ampulla, and discharge of thick, white membranes after sphincterotomy.Video 1

**Fig. 4:**
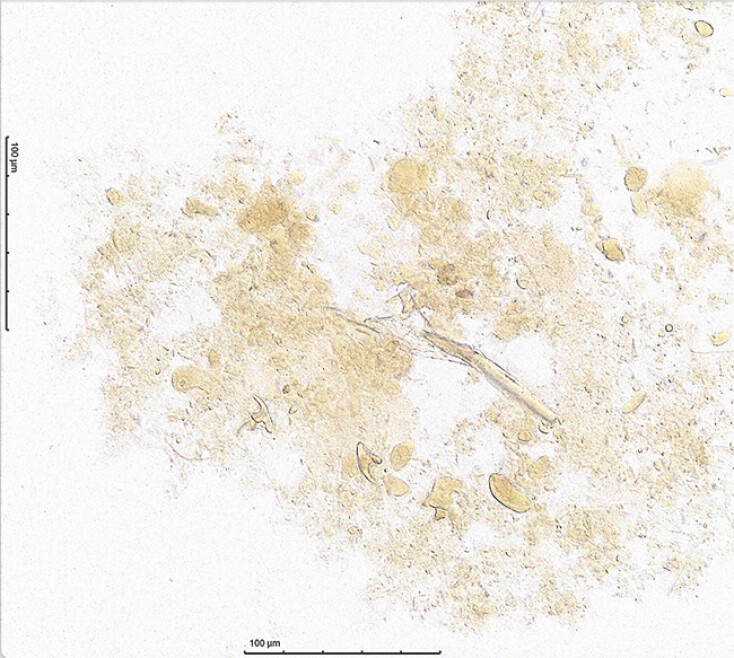
Wet mount iodine microscopy: biliary aspirate with numerous hooklets, confirming echinococcosis.

Evaluation of the biliary aspirate with wet mount iodine microscopy identified numerous hooklets ( Fig. 4 ) and confirmed the diagnosis of a compressive echinococcal cyst. He was first treated with albendazole and then referred to hepatobiliary surgery for resection. Both ERCP and left hepatectomy conferred a significant clinical and biochemical improvement.

A ruptured hydatid cyst can lead to the fish-mouth papilla, which is typically pathognomonic for MD-IPMN. ERCP was effective in the treatment of cholangitis in the context of biliary obstruction from hydatid cyst rupture and biliary aspirate confirmed the diagnosis.

Endoscopy_UCTN_Code_CCL_1AB_2AG_3AD
